# The early asthmatic response is associated with glycolysis, calcium binding and mitochondria activity as revealed by proteomic analysis in rats

**DOI:** 10.1186/1465-9921-11-107

**Published:** 2010-08-06

**Authors:** Yu-Dong Xu, Jian-Mei Cui, Yu Wang, Lei-Miao Yin, Chang-Ke Gao, Yan-Yan Liu, Yong-Qing Yang

**Affiliations:** 1 Yue Yang Hospital, Shanghai University of Traditional Chinese Medicine, Shanghai, China; 2 Molecular Biology Laboratory, Shanghai Research Institute of Acupuncture and Meridian, Shanghai University of Traditional Chinese Medicine, Shanghai, China

## Abstract

**Background:**

The inhalation of allergens by allergic asthmatics results in the early asthmatic response (EAR), which is characterized by acute airway obstruction beginning within a few minutes. The EAR is the earliest indicator of the pathological progression of allergic asthma. Because the molecular mechanism underlying the EAR is not fully defined, this study will contribute to a better understanding of asthma.

**Methods:**

In order to gain insight into the molecular basis of the EAR, we examined changes in protein expression patterns in the lung tissue of asthmatic rats during the EAR using 2-DE/MS-based proteomic techniques. Bioinformatic analysis of the proteomic data was then performed using PPI Spider and KEGG Spider to investigate the underlying molecular mechanism.

**Results:**

In total, 44 differentially expressed protein spots were detected in the 2-DE gels. Of these 44 protein spots, 42 corresponded to 36 unique proteins successfully identified using mass spectrometry. During subsequent bioinformatic analysis, the gene ontology classification, the protein-protein interaction networking and the biological pathway exploration demonstrated that the identified proteins were mainly involved in glycolysis, calcium binding and mitochondrial activity. Using western blot and semi-quantitative RT-PCR, we confirmed the changes in expression of five selected proteins, which further supports our proteomic and bioinformatic analyses.

**Conclusions:**

Our results reveal that the allergen-induced EAR in asthmatic rats is associated with glycolysis, calcium binding and mitochondrial activity, which could establish a functional network in which calcium binding may play a central role in promoting the progression of asthma.

## Background

Asthma is a common yet complex disorder that affects the airways of an estimated 300 million people of all ages. As a widespread disease, asthma places heavy economic burdens on both individuals and society [1]. Despite advances in understanding the pathogenesis of asthma and the development of new anti-asthma therapies, the prevalence and morbidity and mortality rates of the disease have continuously increased worldwide over the past two decades [2]. While the currently available anti-inflammatory and muscle-relaxing drugs are effective in controlling the clinical symptoms, they do not prevent the natural course of asthma [3]. A plausible explanation for this failure is that different biological mechanisms mediate the progression of asthma and asthma symptoms, and unlike the symptoms, asthma progression is insensitive to certain therapies [4]. Therefore, it is necessary to further understand the molecular mechanisms underlying asthma progression to develop new, curative asthma drugs.

The inhalation of allergens by patients with allergic asthma results in the early asthmatic response (EAR). The EAR is characterized by an acute airway obstruction that develops within a few minutes and generally resolves within 1 to 2 h. This is usually followed by the late asthmatic response [5]. As the early phase of the natural course of asthma [4], the EAR is important for the early recognition and treatment of this disease and should receive more attention. Over the past two decades, several animal models that develop the EAR have been reported [6,7]. In our previous study, the EAR was observed 2 to 5 min after provocation in ovalbumin (OVA)-sensitized rats challenged with OVA [8]. Moreover, the pathogenesis of the EAR has been studied by measuring physiologic parameters, variations in serum constituents and by the effect of pretreatment with various drugs [5]. Previous evidence has demonstrated that the EAR can be associated with inflammatory mediators secreted by pulmonary mast cells in an IgE-dependent process, and following the activation of specific receptors, these inflammatory mediators have spasmogenic effects that result in abnormal airway smooth muscle (ASM) contraction [5,9]. These findings have expanded our understanding of the EAR at different levels. However, the molecular mechanism underlying the EAR remains largely unknown. To gain insight into the protein basis of the EAR, a global protein profiling study is urgently needed.

An increasing numbers of proteomics-based asthma studies have been published in recent years, providing useful information about the disease. More importantly, these studies have identified several candidate proteins or novel biomarkers that show an association with the asthma phenotype (an elevated IgE level, bronchial hyperresponsiveness and atopy) [10]. While these findings have furthered our understanding of asthma progression, the present challenge is to understand the natural course of asthma, including the EAR, and to build a functional model to explain the underlying molecular mechanisms of the disease. The primary purpose of this study was to investigate the protein expression patterns in the OVA-induced EAR using proteomic techniques and to elucidate the molecular basis of the EAR.

## Methods

### Animals and sensitization protocol

Pathogen-free, male Sprague-Dawley (SD) rats (4 weeks old, 110-130 g; SLAC Laboratory Animal Co., Shanghai, China) raised in a pathogen-free rodent facility and provided with food and water *ad libitum *were randomly divided into two groups-- control rats (*n *= 15) and asthmatic rats (*n *= 14). The rats were kept in animal facilities approved by the Shanghai Committee for the Accreditation of Laboratory Animals for at least 1 week before the experiments began. The animal experiments conformed to the regulations set forth by the State Science and Technology Commission.

The rats were sensitized and challenged with OVA (grade V, Sigma, Taufkirchen, Germany) according to the protocol described previously [8]. Briefly, on day 0, rats were intraperitoneally injected with 1 mg of OVA precipitated with 10 mg of aluminum hydroxide gel in 1 ml saline. On day 14, allergic rats were anaesthetized with 1% sodium pentobarbitone (wt/vol) at a dose of 50 mg/kg by intraperitoneal injection and then challenged with 1 ml/kg of 5% OVA in saline (5 mg/kg) by injection into the external jugular vein over 10 s. The control rats received the same treatment schedule but were sensitized and challenged with saline instead of OVA.

### Measurement of respiratory function

**Figure 1:**
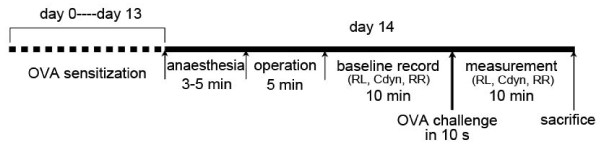
**Protocol and measurements for the rat model of allergic asthma**. Male SD rats were sensitized intraperitoneally at day 0 with 1 mg of OVA precipitated with 10 mg of aluminum hydroxide gel in 1 ml saline. On day 14, rats were anaesthetized with 1% sodium pentobarbitone (wt/vol) at a dose of 50 mg/kg and challenged with 1 ml/kg of 5% OVA in normal saline (5 mg/kg) by injection into the external jugular vein over 10 s. The pulmonary resistance (RL), dynamic compliance (Cdyn) and respiratory rate (RR) were recorded for 10 min before challenge as the baseline values and then immediately measured for another 10 min after the challenge. The changes in the RL, Cdyn and RR were calculated as the differential values subtracted from the corresponding baseline values. The rats were euthanized immediately after the measurements were taken. Control rats were sensitized and challenged with saline instead of OVA.

The technique for the invasive respiratory function measurements used in this study has been described previously [11]. Briefly, a rat was placed in a supine position and warmed with an incandescent lamp after anaesthesia. At the upper part of the trachea, a T-shape incision was made and a T-shape cannula, which was directly attached to a heater-controlled pneumotachograph (Fleisch model 000, Hans Rudolph, USA), was gently inserted into the trachea. Tidal flow was determined by a pneumotachograph connected to a differential pressure transducer (AutoTran, model 600D-011, ± 2 cmH2O). To measure the transpulmonary pressure, a water-filled PE-90 tube was inserted into the esophagus to the level of the mid-thorax (lower one-third of the esophagus) and coupled to a pressure transducer (PT14MX, Jialong Teaching Equipment, Shanghai). The pneumotachograph tidal flow signal was integrated with respect to time to obtain the tidal volume. The pulmonary resistance (RL) and dynamic compliance (Cdyn) were calculated over a complete respiratory cycle using an integration method over flows, volumes and pressures and were continuously recorded with software (Shanghai Medical College, Fudan University) for physiology experiments. The respiratory parameters were averaged in 60-s segments, and the maximum RL, minimum Cdyn and change of respiratory rate (RR) values were taken and calculated as the differential value subtracted from the corresponding baseline value (Figure 1). Following the respiratory function measurements, the rats were euthanized. Lung tissue was excised immediately, dissected from surrounding tissue in ice-cold saline and then immediately frozen in liquid nitrogen.

### Sample preparation

Proteins were extracted from each lung tissue sample using the ReadyPrep Sequential Extraction Kit (Bio-Rad, Hercules, CA) with the following additions to Reagent 1 added just prior to use: PMSF (1 mM), DNase (RNase-free; 20 μg/ml) and RNase (5 μg/ml). The protein concentrations were determined using a modified Bradford assay. For the 2-DE analysis, the protein extracts from each animal in a group were pooled equally according to the protein concentration and stored at -80°C until use.

### Two-dimensional gel electrophoresis (2-DE) and image analysis

A fixed amount of protein (100 μg) was loaded onto 17-cm IPG strips with a linear separation range of pH 3-10 (Bio-Rad, Hercules, CA). Using a Protean IEF Cell (Bio-Rad), the first-dimensional isoelectric focusing (IEF) was performed following the protocol provided by Bio-Rad Laboratories. After equilibration in a buffer (50 mM Tris-HCl pH 8.8, 6 M urea, 20% glycerol and 2% SDS) supplemented with 2% DTT for 15 min and a second incubation in this buffer supplemented with 2.5% iodoacetamide for another 15 min, a Protean II XL cell (Bio-Rad) with a polyacrylamide gel was used for the second-dimensional separation. The proteins in the gel were visualized following silver staining and scanned with a GS-800 densitometer (Bio-Rad). The differentially expressed proteins were detected using PDQuest software version 7.1 (Bio-Rad). Only those spots with at least a threefold change in intensity (*p *< 0.05, *t-*test) were considered differentially expressed.

### Protein identification

Proteins with significant changes in expression were excised from the gels, washed, dehydrated and digested with 12.5 ng/ml trypsin in 0.1 M NH_4_HCO_3_. Liquid-chromatography electrospray-ionization tandem mass spectrometry (LC-ESI-MS/MS) analysis was conducted using a Finnigan LTQ mass spectrometer (ThermoQuest, San Jose, CA) coupled with a Surveyor HPLC system (ThermoQuest). Protein identification using the raw MS/MS data was performed with the SEQUEST software (Thermo Finnigan) by searching against the Swiss-Prot rat protein database. The identification results were filtered based on the Xcorr (1+ ≥ 1.9, 2+ ≥ 2.2, 3+ ≥ 3.75) and the DeltCn (≥ 0.1).

### Bioinformatic analysis

To better understand the key regulated biological processes occurring during the EAR, bioinformatic analysis on the identified proteins, including the gene ontology (GO) classification, the protein-protein interaction (PPI) networking and the biological pathway exploration, were carried out using PPI Spider [12] and KEGG (Kyoto Encyclopedia of Genes and Genomes) Spider [13], which are two freely available web-based tools for the interpretation of experimentally derived protein lists in the context of a global PPI network and a metabolic network, respectively. A dataset containing the standard gene symbols of the identified proteins was uploaded. The significantly enriched GO classes (or terms), PPI networks and biological pathways were determined with the default parameter settings.

### Western blot

The same protein samples used for the 2-DE were analyzed, and the polyvinylidene fluoride membranes were blocked overnight in a PBS-Tween buffer with 5% nonfat milk. The membranes were then probed using antibodies specific for ERp29 (endoplasmic reticulum protein ERp29), RhoGDI2 (Rho, GDP dissociation inhibitor beta), S100A8 or β-actin in the blocking buffer. The staining was visualized using ECL reagents (Beyotime, Haimen, China). The densitometric evaluation was carried out using Quantity One software version 4.6 (Bio-Rad), and the relative quantity of an individual protein is expressed as the ratio of the gray scale respective to that of β-actin.

### Semi-quantitative RT-PCR

RNA was extracted using TRIzol reagent (Invitrogen, Carlsbad, CA) and reverse transcribed using the RevertAid First Strand cDNA Synthesis Kit (MBI Fermentas, Vilnius, Lithuania). PCR amplification was conducted using the TaKaRa RNA PCR Kit (TaKaRa, Dalian, China). The primers used were specific for S100A8, S100A11, VDAC1 (voltage-dependent anion channel 1) or β-actin (see additional file 1). The PCR products were resolved by agarose gel electrophoresis and analyzed using Quantity One software (Bio-Rad). The relative mRNA level of each individual gene is presented as the expression ratio with respect to the housekeeping gene β-actin.

### Statistical analysis

Student's *t*-test was performed to evaluate the differences between the control and asthmatic group when the data were normally distributed, while the Mann-Whitney test was used when the data were not normally distributed. The differences among continuous data in each group obtained from the respiratory function measurement were assessed with the Kruskal-Wallis test. The statistical analysis for PPI Spider and KEGG Spider was performed using the Monte Carlo simulation procedure. A *p *value less than 0.05 was considered statistically significant.

## Results

### Respiratory function measurement following OVA challenge

**Figure 2:**
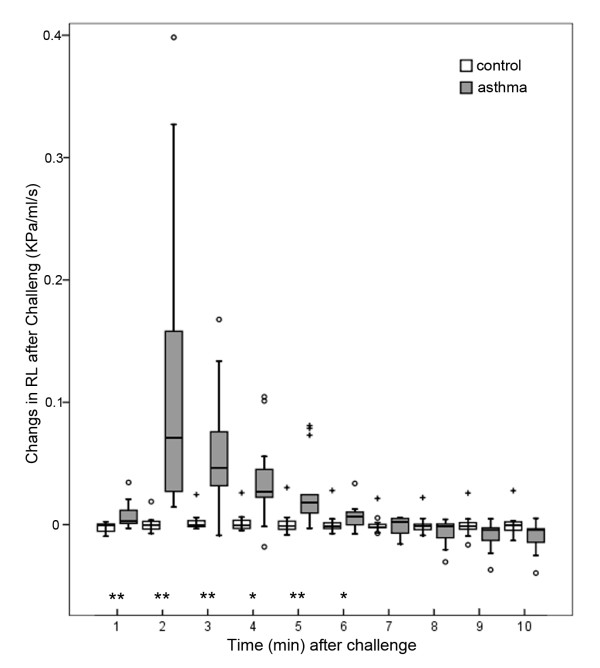
**Changes in the pulmonary resistance after challenge**. Box plots of the pulmonary resistance (RL) within 10 min after challenge in asthmatic rats (*n *= 14, sensitized and challenged with OVA) and controls (*n *= 15, sensitized and challenged with normal saline). The RL values are expressed as differential values subtracted from the corresponding baseline values (Figure 1). Mild outliers (○) are cases with values between 1.5 and 3 box lengths from the upper or lower edge of the box. Extreme outliers (+) are cases with values more than 3 box lengths from the upper or lower edge of the box. As measured using the Kruskal-Wallis test, the differences in the RL at each time point in the asthmatic group, but not in the control group, were significant (*p *< 0.01). Significant differences in the RL were observed between the two groups at 1-6 min (* *p *< 0.05, ** *p *< 0.01, Mann-Whitney test) with a maximum increase in asthmatic rats observed 2 min after challenge.

The OVA challenge in sensitized rats induced a significant increase in the RL and, simultaneously, significant decreases in the Cdyn and RR compared with control rats. These alterations indicated an OVA-induced EAR. The most prominent change in asthmatic rats was in the RL with a maximum increase of 0.110 ± 0.115 kPa/ml/s (mean ± SD, *p *< 0.01, compared with the controls) 2 min after provocation (Figure 2). When compared with controls, the Cdyn showed a maximum decrease at 3 min, and the RR showed a maximum decrease at 2 min (data not shown).

### Proteomic analysis

**Figure 3:**
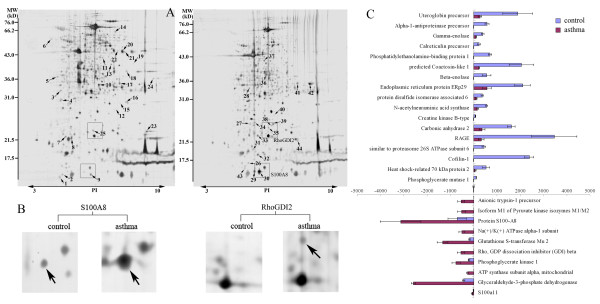
**Protein expression profiles in lung and asthmatic lung tissue**. (A) Representative 2-D gel images of proteins isolated from normal control (left) and asthmatic rats (right). Total protein extracts were separated on 17-cm linear IPG strips (pH 3-10) in the first dimension followed by 12% SDS-PAGE in the second dimension and visualized by silver staining. (Left) The 25 down-regulated protein spots in asthmatic rats are marked with arrows. (Right) The 19 up-regulated protein spots in asthmatic rats are marked with arrows. The numbers correspond to the spot identification numbers listed in Table 1. The molecular weight standards and the pH range are shown at the left and the bottom of the gels, respectively. (B) Cropped 2-DE gel images of the S100A8 and RhoGDI2 protein spots. The selected area was symmetrically boxed, and the arrows indicate each protein spot or its theoretical location. (C) The expression profile of 28 most significantly changed proteins that were detected more than three times. The upper portion shows the 17 proteins down-regulated in asthmatic lung tissue, and the lower portion shows the 11 proteins that were up-regulated. Each spot volume was quantified from the intensity of the spots using PDQuest software. The bars represent the mean ± SD from three replicated 2-DE gels. The information for each altered spot is reported in Table 1.

The protein in the sample from each group was measured at least three times and verified to ensure that the same protein patterns were obtained. In general, we detected approximately 550-650 protein spots in each 2-DE gel and observed a high rate of overlap (>90%) in the parallel gels. In total, 44 protein spots showed consistently differential expression between the asthmatic group and the control in all three parallel experiments, with 25 down-regulated spots and 19 up-regulated spots (Figure 3A). After identification via mass spectrometry, 42 protein spots corresponding to 36 unique proteins were successfully identified. Of the unique proteins, 21 were down-regulated and 16 were up-regulated in the asthmatic rats (Table 1, Figure 3C). For the tubulin alpha-1A chain, both up- and down-regulated expression patterns were observed, possibly reflecting differential post-translational modification of the same protein.

**Table 1:** Differentially expressed proteins in the early asthmatic response

Spot*	Protein Name	Gene Symbol	Swiss-Prot/RefSeq accession No.	**Theoretical Mr (kD)/pI**^**†**^	**Pept./% Seq.Cov**^**‡**^
**Down-regulated in Asthmatic rats**					
1	Uteroglobin precursor	Scgb1a1	P17559	10.4/4.9	7/25.00%
2	Alpha-1-antiproteinase precursor	Serpina1	P17475	46.1/5.7	6/11.68%
3	Tubulin alpha-1A chain	Tuba1a	P68370	50.1/4.9	6/9.31%
4	Gamma-enolase	Eno2	P07323	47.0/7.0	2/5.07%
5	Heat shock cognate 71 kDa protein	Hspa8	P63018	70.8/5.4	3/4.18%
6	Calreticulin	Calr	P18418	48.0/4.3	49/36.06%
7	Phosphatidylethanolamine-binding protein 1	Pebp1	P31044	20.8/5.5	9/25.13%
8	predicted Coactosin-like 1	Cotl1	B0BNA5	15.9/5.3	6/27.46%
9	Beta-enolase	Eno3	P15429	47.0/7.0	2/5.07%
10	Endoplasmic reticulum protein ERp29 precursor	Erp29	P52555	28.6/6.2	71/48.85%
11	protein disulfide isomerase associated 6	Pdia6	Q63081	48.8/5.0	20/26.07%
12	Beta-enolase	Eno3	P15429	47.0/7.0	3/5.07%
13	N-acetylneuraminic acid synthase	Nans	B1WC26	40.0/6.4	9/17.83%
14	Bifunctional purine biosynthesis protein PURH	Atic	O35567	64.2/6.7	5/6.42%
15	Creatine kinase B-type	Ckb	P07335	42.7/5.4	2/4.46%
16	Isoform 1 of Fibrinogen beta chain precursor	Fgb	P14480-1	54.2/7.9	55/45.30%
17	Carbonic anhydrase 2	Ca2	P27139	29.1/6.9	32/50.38%
18	22 kDa protein	--	--	--	5/22.12%
19	Heat shock cognate 71 kDa protein	Hspa8	P63018	70.8/5.4	19/18.89%
20	Advanced glycosylation end product-specific receptor precursor	Ager	Q63495	42.7/5.8	25/23.38%
21	similar to proteasome 26 S ATPase subunit 6	Psmc6	Q32PW9	45.8/7.6	17/27.79%
22	22 kDa protein	--	--	--	7/25.00%
23	Cofilin-1	Cfl1	P45592	18.5/8.2	95/78.92%
24	Heat shock-related 70 kDa protein 2	Hspa2	P14659	69.6/5.5	6/4.27%
25	Phosphoglycerate mutase 1	Pgam1	P25113	28.8/6.7	11/16.93%
**Up-regulated in Asthmatic rats**					
26	Myotrophin	Mtpn	P62775	12.9/5.3	6/25.42%
27	Anionic trypsin-1 precursor	Prss1	P00762	26.0/4.7	4/8.13%
28	Isoform M1 of Pyruvate kinase isozymes M1/M2	Pkm2	P11980	58.0/6.6	40/25.52%
29	Protein S100-A8	S100a8	P50115	10.2/5.7	6/56.18%
30	Protein S100-A8	S100a8	P50115	10.2/5.7	26/41.57%
31	Sodium/potassium-transporting ATPase subunit alpha-1 precursor	Atp1a1	P06685	11.3/5.3	16/13.29%
32	similar to destrin	LOC296197	XP_215862	18.5/7.5	11/27.88%
33	Glutathione S-transferase Mu 2	Gstm2	P08010	25.7/6.9	18/38.99%
34	Tubulin alpha-1A chain	Tuba1a	P68370	50.1/4.9	11/13.53%
35	Rho, GDP dissociation inhibitor (GDI) beta	Arhgdib	Q5M860	22.9/5.0	4/21.00%
36	Serum albumin precursor	Alb	P02770	68.7/6.1	194/60.36%
37	Phosphoglycerate kinase 1	Pgk1	P16617	44.5/8.0	9/29.50%
38	Actin, alpha skeletal muscle	Acta1	P68136	42.0/5.2	8/11.94%
39	Serum albumin precursor	Alb	P02770	68.7/6.1	7/6.58%
40	ATP synthase subunit alpha, mitochondrial precursor	Atp5a1	P15999	59.8/6.2	53/22.24%
41	Glyceraldehyde-3-phosphate dehydrogenase	Gapdh	P04797	35.8/8.1	5/12.31%
42	Glyceraldehyde-3-phosphate dehydrogenase	Gapdh	P04797	35.8/8.1	17/29.73%
43	S100a11	S100a11	Q6B345	11.1/5.6	3/26.5%
44	similar to Ras suppressor protein 1	Rsu1	XP_001057090	31.5/8.5	4/10.83%

The proteins were identified using LC-ESI-MS/MS following in-gel tryptic digest. Proteins are designated with their protein name, standard gene symbol and Swiss-Prot/NCBI RefSeq accession number.

* The spot number from 2-D gel analysis (Figure 3).

† The theoretical molecular mass (Mr) and isoelectric point (pI) of the identified protein.

‡ The number of matching peptides (Pept.) and the percentage of the total amino acid sequence covered by those peptides (% Seq.Cov) from the mass mapping experiments.

### Bioinformatic analysis

**Figure 4:**
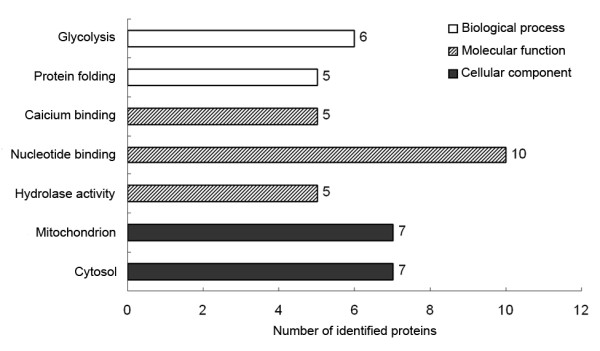
**Gene Ontology (GO) classification**. GO classification for all of the identified proteins based on three domains: biological process, molecular function and cellular component. The number of proteins enriched in each GO class is shown at the right side of the bars.

The gene ontology (GO) provides three structured terms to describe gene products: (i) the biological process to which a gene product contributes; (ii) the molecular function that describes the biochemical activities of the gene product; and (iii) the cellular component, which refers to the subcellular location where a gene product is active and occurs [14]. In this study, the GO classification was performed to identify the most important GO classes (or terms) (*p *< 0.05, Monte Carlo simulation procedure) of the input proteins (Figure 4, additional file 2). "Glycolysis" was a significantly enriched GO class of biological process category involving six proteins, which indicates a possible role for glycolysis during the EAR. Among the enriched GO classes of molecular function category, "calcium ion binding," which involved five proteins, was the most attractive given the potential role of Ca^2+ ^and Ca^2+ ^homeostasis in airway reactivity and asthma. In the cellular component category, seven proteins were classified into the GO class "mitochondrion," which is probably due to the mitochondrial activation during the EAR.

**Figure 5:**
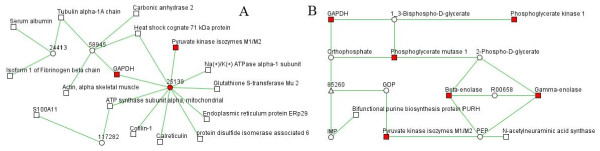
**The *in silico *networks inferred by PPI Spider and KEGG Spider**. The proteins identified in the proteomic study are marked by rectangles, and red nodes represent the proteins involved in glycolysis. (A) The PPI Spider network model covers 16 identified proteins. The intermediate proteins not detected in the proteomic study are marked by circles and the corresponding Entrez gene IDs: 25139, GLUT4 (solute carrier family 2 [facilitated glucose transporter], member 4); 24413, NR3C1 (glucocorticoid receptor); 58945, DYNLL1 (dynein light chain 1, cytoplasmic); and 117282, hnRNP K (heterogeneous nuclear ribonucleoprotein K). (B) The KEGG Spider network model covers eight identified proteins. The intermediate proteins not detected in the proteomic study are marked by triangles and the corresponding Entrez gene ID: 85260, ENTPD6 (ectonucleoside triphosphate diphosphohydrolase 6). The chemical compounds involved are marked with circles.

A protein-protein interaction (PPI) network created by PPI Spider covered 16 identified proteins (Figure 5A). Notably, ten proteins were connected by the intermediate protein GLUT4 (solute carrier family 2 [facilitated glucose transporter], member 4), which promotes the transport of glucose from the blood to target tissues [15]. The pathway network constructed by KEGG Spider covered eight identified proteins, and a total of six proteins were involved in the "glycolysis" pathway constituting the key nodes of this network (Figure 5B). The inferred *in silico *networks further suggest that glucose metabolism, especially glycolysis, is involved in the pathogenesis of the EAR.

### Western blot analysis of RhoGDI2, ERp29 and S100A8

**Figure 6:**
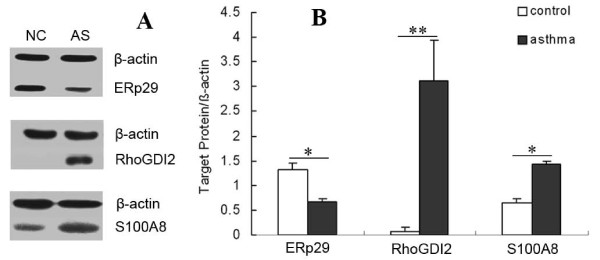
**Validation of the 2-DE proteomic data by western blot analysis**. (A) A representative western blot visualizing the ERp29, RhoGDI2 and S100A8 expression levels. β-actin was used to demonstrate equal loading. NC = normal control group (*n *= 15) and AS = asthmatic group (*n *= 14). (B) The densitometric quantification of individual protein is expressed as the fold change compared to β-actin. The data are presented as the mean ± SD of triplicate experiments, and similar results were seen in all experiments. Statistical comparisons were made using Student's *t*-test. *p < 0.05, **p < 0.01 comparing the asthmatic group with the control group. The expression pattern of these proteins is in agreement with the 2-DE results.

To verify the 2-DE findings, we further examined the expression of RhoGDI2, ERp29 and S100A8 using western blots. Compared with the controls, the expression levels of RhoGDI2 and S100A8 were significantly increased (*p *< 0.01 and *p *< 0.05, respectively) in the asthmatic rats, whereas the expression of ERp29 was significantly decreased (*p *< 0.01) in the asthmatic rats (Figure 6). These expression patterns were consistent with the patterns detected in the 2-DE analysis. These results confirm the reliability of this comparative proteomic study.

### mRNA Level of S100A8, S100A11 and VDAC1

**Figure 7:**
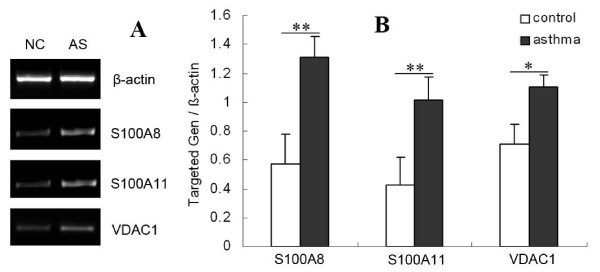
**Semi-quantitative RT-PCR analysis of the S100A8, S100A11 and VDAC1 mRNA expression level**. (A) Representative RT-PCR visualizing the S100A8, S100A11 and VDAC1 mRNA expression levels; β-actin was used as an internal control. NC = normal control group (*n *= 15) and AS = asthmatic group (*n *= 14). (B) The densitometric quantification of the mRNA level of the individual genes is expressed as the fold change compared to β-actin. The data are presented as the mean ± SD from triplicate experiments, and similar results were seen in all experiments. Statistical comparisons were made using Student's *t*-test. *p < 0.05, **p < 0.01 comparing the asthmatic group with the control group.

The mRNA level of S100A8 and S100A11 was low in the control group, and this expression was significantly higher in the asthmatic group (both *p *< 0.01, Figure 7). These results not only demonstrate the up-regulation of S100A8 and S100A11 at the mRNA level, but also suggest enhanced Ca^2+ ^binding during the EAR.

To assess whether the mitochondrial activity was increased during the EAR, the mRNA expression pattern of VDAC1, an outer membrane mitochondrial protein, was investigated using RT-PCR. As seen in Figure 7, the mRNA level of VDAC1 was significantly up-regulated in the lungs of asthmatic rats compared to the controls (*p *< 0.01).

## Discussion

The EAR is the early phase of the natural course of allergic asthma and is important for the early recognition and treatment of this disease. Although a number of proteomic studies have identified several candidate proteins involved in the pathogenesis of asthma, few proteomic studies of the EAR have been reported [10]. In this study, we compared the protein profiles of the lung tissue extracted from rats with an OVA-induced EAR with those of the controls using 2-DE/MS-based proteomic analysis. We identified 44 differentially expressed protein spots corresponding to 36 unique proteins. These proteins reflected a protein profile that changed during the disease process, which included sensitization, allergen challenge and the subsequent EAR. A functional analysis of these changed proteins revealed some possible key molecular mechanisms underlying the EAR.

### Glycolysis during the EAR

Glycolysis is a fundamental system for energy metabolism in most organisms, and glycolytic enzymes are present in all mammalian cells and tissues. Over the past few decades, several lines of evidence have indicated that the EAR-associated events, including hypoxia and ASM contraction, could affect and be affected by energy and glucose metabolism [16-18]. Using a proteomic approach, our study demonstrates that the EAR is closely associated with glycolysis.

The GO analysis demonstrated that six of the identified proteins are important in glycolysis (Figure 4). Among them, phosphoglycerate kinase 1 is well known as the seventh enzyme of the glycolytic pathway, which catalyzes the reversible conversion of 1,3-diphosphoglycerate to 3-phosphoglyceric acid and a single ATP molecule. It has been recently reported that phosphoglycerate kinase 1 can enhance the glycolytic capacity of an organ and support the maintenance of cellular energetics in wood frogs with hypoxia [19]. Pyruvate kinase catalyzes the penultimate step in glycolysis, where it transfers phosphate from phosphoenolpyruvate to ADP, leading to the formation of ATP and pyruvate. The energy regeneration by pyruvate kinase is independent of the oxygen supply, which allows for the survival of cells under hypoxic conditions [20]. Glyceraldehyde-3-phosphate dehydrogenase is another essential glycolytic enzyme expressed in all prokaryotic and eukaryotic organisms. Its role is to convert glyceraldehyde-3-phosphate to 1,3-diphosphoglycerate during glycolysis. The significantly higher expression level of all of the above proteins in asthmatic rats suggests the up-regulation of glycolysis during the EAR. Two other glycolytic enzymes, beta-enolase and phosphoglycerate mutase, both found in the lung tissue proteome, are also rate-controlling enzymes in glycolysis. Their decreased expression could regulate the rate of glycolysis as well as the ATP-dependent reactions involved in the EAR. Our proteomic analysis indicates that glycolysis is a key regulatory biological process in the pathogenesis of the EAR. These glycolytic enzymes may facilitate the anaerobic production of ATP to meet the increasing cellular energy demands during ASM contraction in the hypoxic conditions induced by airway obstruction. It should be noted, however, that glycolysis alone may not meet the needs of the contractions because it only provides two ATP molecules from every glucose molecule while oxidative phosphorylation and lactic acid may be more efficient energy-supplying processes under hypoxic conditions. However, our results confirm the importance of energy metabolism and energy balance during the EAR, which could provide new clues for the further study of asthma.

Additionally, a recent review reported that some glycolytic enzymes are complicated, multifaceted proteins rather than simple components of the glycolytic pathway [21]. These enzymes also possessed non-glycolytic functions involved in transcriptional regulation, the stimulation of cell motility and the regulation of apoptosis. With these multifaceted functions, glycolytic enzymes could play a role in the hyperplasia or hypertrophy of ASM cells in asthma disease. Therefore, the identification of differentially expressed glycolytic proteins in the lung tissue proteome also suggests that airway remolding may develop during the EAR.

### Calcium binding during the EAR

Calcium binding is a key initiating process for the EAR because the binding of Ca^2+ ^to calmodulin to form the Ca^2+^/calmodulin complex results in the activation of myosin light-chain kinase, which, in turn, mediates ASM contraction [22]. In this study, we observed the increased expression of Ca^2+^-binding proteins (CaBPs) in the 2-DE analysis and further verified the increased expression of S100A8 and S100A11 using western blot and RT-PCR, respectively. In our previous study, we also reported the increased mRNA level of another CaBP, S100A9, through the serial analysis of gene expression during the EAR [8].

Calcium is a central player that mediates various pathophysiological reactions through binding with CaBPs. Cells contain classes of CaBPs that regulate the level of cytosolic Ca^2+ ^and transduce intracellular Ca^2+^-mediated signals. S100 proteins, which are highly homologous, low-molecular weight (10-12 kDa), acidic proteins, constitute one of the CaBP families. They are believed to be involved in the regulation of Ca^2+ ^homeostasis, smooth muscle contraction and other cellular processes through their binding to Ca^2+ ^and interactions with various effector proteins [23]. S100 proteins have received increased attention in the study of asthma due to their close association with inflammation [24]. S100A8 and A9, also known as calgranulins, have been shown to play a role in the initiation and regulation of chronic and acute inflammatory diseases, including asthma [24,25]. A study examining human asthmatic bronchoalveolar lavage fluid showed that elevated levels of S100A8 and S100A9 were strongly linked to allergic inflammation [26]. Moreover, the inhibition of S100A8 and S100A9 could reduce the migration of inflammatory cells into the lungs in a mouse model of asthma [27]. Recent evidence has also demonstrated that S100A8 and S100A9 are involved in a novel pro-inflammatory signaling pathway in which they contribute to the activation of central cellular pathways, including the p38 or p44/42 MAP kinases and NF-κB signaling components, which subsequently induce the expression of many pro-inflammatory molecules [28,29]. In our studies, elevated expression levels of S100A8 and S100A9 were found in asthmatic rats, indicating that during the EAR, CaBPs such as S100A8 and A9 could trigger the asthmatic inflammatory response.

Calreticulin and protein disulfide-isomerase A6 are endoplasmic reticulum-resident, luminal Ca^2+^-buffering chaperones involved in the storage of Ca^2+ ^and the regulation of the intracellular Ca^2+ ^concentration ([Ca^2+^]_i_) [30,31]. Their decreased expression levels suggest that the release of Ca^2+ ^from the intracellular Ca^2+ ^stores causes an elevation of the [Ca^2+^]_i _in ASM cells. ERp29 is a newly identified ER lumen-resident protein that lacks Ca^2+ ^binding activities. In our study, decreased ERp29 expression was detected during the EAR, and this change in expression was validated by western blot. Although ERp29 cannot bind Ca^2+^, it may interact with Ca^2+^-binding molecular chaperones on the membrane of the mitochondrial sheath and thus may indirectly influence Ca^2+ ^transport [32]. S100A11 is a CaBP with an intermediate expression level in lung tissue and plays a key role in the regulation of enzymatic activity, cell growth, apoptosis and inflammation [33]. In contrast to the proteomic study of a chronic mouse asthma model that demonstrated marked down-regulation of S100A11 [34], we observed an increased expression level of S100A11 during the EAR. However, the precise role of this protein in asthma remains unclear and needs further investigation.

It has long been established that an increased [Ca^2+^]i might account for the heightened ASM contractility, increased mucous gland secretion and elevated mast cell mediator secretion rate observed in asthma [35]. Consequently, agonist-induced Ca^2+ ^signaling and altered Ca^2+ ^homeostasis have been regarded as possible therapeutic targets in the pathophysiology of asthma. It is highly plausible that the inhibition of Ca^2+ ^mobilization into ASM cells could effectively reverse acute airway obstruction. Based on the results presented in this study, Ca^2+ ^and its role in asthma should be examined in more detail. Compared with Ca^2+ ^mobilization, the binding of Ca^2+ ^and the downstream biological events induced by the interaction between CaBPs and effector proteins may play a more crucial role in the progression of asthma. Further studies on Ca^2+ ^binding and its effects will help determine whether CaBPs can be used as novel drug targets for the treatment of asthma.

### Mitochondrial activity during the EAR

Mitochondria have a critical role in ATP production and are sometimes described as "cellular power plants" [36]. In addition to their bioenergetic function, mitochondria have been increasingly recognized as key players in cellular regulatory systems including Ca^2+ ^handling and apoptosis, as well as numerous other catabolic and anabolic pathways [37,38]. Accumulating clinical and experimental evidence has suggested a possible role for mitochondrial activity in the pathogenesis of asthma. In asthmatic ASM cells, there was a higher basal energy turnover due to the increased number and activity of mitochondria [39]. Similarly, Ca^2+^-dependent enhanced mitochondrial biogenesis has been demonstrated in the bronchial smooth muscle of asthmatic patients, and this was causatively linked to airway remodeling [40].

In our study, seven of the identified proteins were enriched in the GO class "mitochondrion" (Figure 4). Among them was Atp5a1, which is a well-characterized mitochondrial protein found in the mitochondrial inner membrane [41]. It is well known that Atp5a1 is an important, enzymatically active member of the F0-F1 mitochondrial ATP synthase (Complex V). This complex is the final electron acceptor in the mitochondrial electron transport chain necessary for the generation of ATP. The enhanced expression of Atp5a1 further illustrates the energetic adaptations in the ASM with increased contractility during the EAR. Glyceraldehyde-3-phosphate dehydrogenase and pyruvate kinase are also mitochondria-localized proteins that were up-regulated in the lung proteome of asthmatic rats. Previous evidence has shown that these glycolytic enzymes can be attached to the outside of mitochondria, where they funnel pyruvate directly into the citric acid cycle in response to cellular energy demands [42,43].

To further investigate whether these mitochondrial proteins are linked to the relative mitochondrial abundance, we examined the mRNA expression pattern of VDAC1, a mitochondrial protein localized to the outer mitochondrial membrane that is commonly used to assess the mitochondrial mass and activity in a specific sample [40]. The significantly up-regulated mRNA level of VDAC1 in the asthmatic group further indicates the elevated biological activity of mitochondria during the EAR. Our findings suggest that mitochondria may represent a potential target for the treatment of asthma in the early phase. Further studies are required to determine the exact functional significance of mitochondria in asthma and assess whether drugs interfering with mitochondrial activity can prevent and/or reverse asthma progression.

In general, the contractility of ASM during the EAR requires an elevation in the [Ca^2+^]_i_. Mitochondria can transiently store Ca^2+^, and together with the ER, can function as the main source of the Ca^2+ ^that is released to increase the [Ca^2+^]_i _[44]. In addition to initiating ASM contraction by binding with calmodulin, the increased [Ca^2+^]_i _could facilitate the binding of Ca^2+ ^to Ca^2+ ^signaling proteins, such as the S100 proteins. This binding would, in turn, allow their interaction with a wide spectrum of effector proteins to activate Ca^2+ ^signaling pathways in ASM cells. Increased glycolysis and mitochondrial activity during the EAR would ensure an adequate energy supply necessary for ASM contraction, Ca^2+ ^binding, transport and the transduction of Ca^2+ ^signaling under hypoxic conditions through an interactive process. Taken together, a multifaceted picture emerges from our proteomic study. It suggests key roles for cellular energy adaptation and the propagation of Ca^2+ ^signaling during the EAR in which Ca^2+ ^binding may play a central role to promote the progression of asthma.

## Conclusions

In this study, we used a 2-DE/MS-based proteomic approach to examine the lung protein profile of asthmatic rats during the EAR. A comparison of the asthmatic rats with the normal controls revealed differences in the protein expression pattern that were highlighted by the gene ontology classification, the PPI interaction networking and the biological pathway exploration. Our results indicate that the allergen-induced EAR is associated with glycolysis, Ca^2+ ^binding and mitochondrial activity, which could establish a functional network with Ca^2+ ^binding as the central player to promote the progression of asthma. These findings may provide new clues important for to the early recognition and treatment of asthma.

## Competing interests

The authors declare that they have no competing interests.

## Authors' contributions

YYQ designed the experiments and drafted and revised the manuscript. XYD participated in the study design, performed the 2-DE/MS experiments, bioinformatic analysis, RT-PCR, western blot, data analysis and drafted the manuscript. CJM duplicated the rat asthmatic model and prepared samples for the proteomic study. WY and YLM participated in the study design, 2-DE/MS experiments and the drafting of the manuscript. GCK participated in the animal experiments. LYY participated in the review of the manuscript. All authors read and approved the final manuscript.

## Supplementary Material

Additional file 1 **Primer sequences for the selected genes and the semi-quantitative RT-PCR conditions**. This table lists the primer sequences used to amplify the three genes of interest (S100A8, S100A11 and VDAC1) and the reference gene (β-actin) as well as the corresponding semi-quantitative RT-PCR conditions. Click here for file

Additional file 2 **The list of the differentially expressed proteins in the enriched gene ontology (GO) classes**. This table lists the details of the GO classification including the enriched GO classes and corresponding differentially expressed proteins. Click here for file

## References

[B1] Bahadori K Doyle-Waters MM Marra C Lynd L Alasaly K Swiston J FitzGerald JM Economic burden of asthma: a systematic review BMC Pulm Med 2009 9 24 10.1186/1471-2466-9-24 19454036 PMC2698859

[B2] Braman SS The global burden of asthma Chest 2006 130 4S 12S 10.1378/chest.130.1_suppl.4S 16840363

[B3] Fang C Corrigan CJ Ying S The treatment targets of asthma: from laboratory to clinic Inflamm Allergy Drug Targets 2008 7 119 128 10.2174/187152808785107624 18691142

[B4] Martinez FD Inhaled corticosteroids and asthma prevention Lancet 2006 368 708 710 10.1016/S0140-6736(06)69261-1 16935667

[B5] Weersink EJ Postma DS Aalbers R de Monchy JG Early and late asthmatic reaction after allergen challenge Respir Med 1994 88 103 114 10.1016/0954-6111(94)90021-3 8146407

[B6] Takeda H Kogame A Tanaka H Nagai H Time course study for airway inflammation and responsiveness by repeated provocation of aeroantigen in guinea pigs Prostaglandins 1997 54 805 820 10.1016/S0090-6980(97)00158-5 9491210

[B7] Cieslewicz G Tomkinson A Adler A Duez C Schwarze J Takeda K Larson KA Lee JJ Irvin CG Gelfand EW The late, but not early, asthmatic response is dependent on IL-5 and correlates with eosinophil infiltration J Clin Invest 1999 104 301 308 10.1172/JCI7010 10430611 PMC408423

[B8] Yin LM Jiang GH Wang Y Liu YY Jin WR Zhang Z Xu YD Yang YQ Serial analysis of gene expression in a rat lung model of asthma Respirology 2008 13 972 982 18922145 10.1111/j.1440-1843.2008.01398.x

[B9] Fahy JV Fick RB, Jardieu PM Pathophysiology of the Airway Response to Inhaled Allergen in Asthmatic Subjects: Role of IgE IgE and Anti-IgE Therapy in Asthma and Allergic Disease 2002 219 233

[B10] Osei-Kumah A Hodyl N Clifton VL Proteomics in asthma Expert Review of Clinical Immunology 2008 4 713 721 10.1586/1744666X.4.6.713 20477121

[B11] Yin LM Jiang GH Wang Y Liu YY Jin WR Xu YD Zhang QH Yang YQ Use of serial analysis of gene expression to reveal the specific regulation of gene expression profile in asthmatic rats treated by acupuncture J Biomed Sci 2009 16 46 19419550 10.1186/1423-0127-16-46 PMC2698896

[B12] Antonov AV Dietmann S Rodchenkov I Mewes HW PPI spider: a tool for the interpretation of proteomics data in the context of protein-protein interaction networks Proteomics 2009 9 2740 2749 10.1002/pmic.200800612 19405022

[B13] Antonov AV Dietmann S Mewes HW KEGG spider: interpretation of genomics data in the context of the global gene metabolic network Genome Biol 2008 9 R179 10.1186/gb-2008-9-12-r179 19094223 PMC2646283

[B14] Dimmer EC Huntley RP Barrell DG Binns D Draghici S Camon EB Hubank M Talmud PJ Apweiler R Lovering RC The Gene Ontology - Providing a Functional Role in Proteomic Studies Proteomics 2008 8 NA 10.1002/pmic.200800002

[B15] Kandror KV A long search for Glut4 activation Sci STKE 2003 2003 PE5 10.1126/stke.2003.169.pe5 12582199

[B16] Kroeger E Stephens NL Effect of hypoxia on energy and calcium metabolism in airway smooth muscle Am J Physiol 1971 220 1199 1204 5574637 10.1152/ajplegacy.1971.220.5.1199

[B17] Souhrada JF Loader J Role of glucose in contractility of airway smooth muscle Respir Physiol 1979 36 231 247 10.1016/0034-5687(79)90027-6 441576

[B18] Zhao W Guenard H Bronchial smooth muscle energetics: effect of iodoacetate and hypoxia Respir Physiol 1994 96 285 296 10.1016/0034-5687(94)90133-3 8059090

[B19] Wu S Storey JM Storey KB Phosphoglycerate kinase 1 expression responds to freezing, anoxia, and dehydration stresses in the freeze tolerant wood frog, Rana sylvatica J Exp Zool Part A Ecol Genet Physiol 2009 311 57 67 10.1002/jez.495 18785212

[B20] Mazurek S Boschek CB Hugo F Eigenbrodt E Pyruvate kinase type M2 and its role in tumor growth and spreading Semin Cancer Biol 2005 15 300 308 10.1016/j.semcancer.2005.04.009 15908230

[B21] Kim JW Dang CV Multifaceted roles of glycolytic enzymes Trends Biochem Sci 2005 30 142 150 10.1016/j.tibs.2005.01.005 15752986

[B22] Stephens NL Cheng ZQ Fust A Sensitized airway smooth muscle plasticity and hyperreactivity: a review Can J Physiol Pharmacol 2007 85 679 685 10.1139/Y07-061 17823632

[B23] Santamaria-Kisiel L Rintala-Dempsey AC Shaw GS Calcium-dependent and -independent interactions of the S100 protein family Biochem J 2006 396 201 214 10.1042/BJ20060195 16683912 PMC1462724

[B24] Lorenz E Muhlebach MS Tessier PA Alexis NE Duncan Hite R Seeds MC Peden DB Meredith W Different expression ratio of S100A8/A9 and S100A12 in acute and chronic lung diseases Respir Med 2008 102 567 573 10.1016/j.rmed.2007.11.011 18164192 PMC2347354

[B25] Nacken W Roth J Sorg C Kerkhoff C S100A9/S100A8: Myeloid representatives of the S100 protein family as prominent players in innate immunity Microsc Res Tech 2003 60 569 580 10.1002/jemt.10299 12645005

[B26] Wu J Kobayashi M Sousa EA Liu W Cai J Goldman SJ Dorner AJ Projan SJ Kavuru MS Qiu Y Thomassen MJ Differential proteomic analysis of bronchoalveolar lavage fluid in asthmatics following segmental antigen challenge Mol Cell Proteomics 2005 4 1251 1264 10.1074/mcp.M500041-MCP200 15951573

[B27] Greenlee KJ Corry DB Engler DA Matsunami RK Tessier P Cook RG Werb Z Kheradmand F Proteomic identification of in vivo substrates for matrix metalloproteinases 2 and 9 reveals a mechanism for resolution of inflammation J Immunol 2006 177 7312 7321 17082650 10.4049/jimmunol.177.10.7312 PMC2580826

[B28] Sunahori K Yamamura M Yamana J Takasugi K Kawashima M Yamamoto H Chazin WJ Nakatani Y Yui S Makino H The S100A8/A9 heterodimer amplifies proinflammatory cytokine production by macrophages via activation of nuclear factor kappa B and p38 mitogen-activated protein kinase in rheumatoid arthritis Arthritis Res Ther 2006 8 R69 10.1186/ar1939 16613612 PMC1526633

[B29] Hermani A De Servi B Medunjanin S Tessier PA Mayer D S100A8 and S100A9 activate MAP kinase and NF-kappaB signaling pathways and trigger translocation of RAGE in human prostate cancer cells Exp Cell Res 2006 312 184 197 10.1016/j.yexcr.2005.10.013 16297907

[B30] Michalak M Groenendyk J Szabo E Gold LI Opas M Calreticulin, a multi-process calcium-buffering chaperone of the endoplasmic reticulum Biochem J 2009 417 651 666 10.1042/BJ20081847 19133842

[B31] Fullekrug J Sonnichsen B Wunsch U Arseven K Nguyen Van P Soling HD Mieskes G CaBP1, a calcium binding protein of the thioredoxin family, is a resident KDEL protein of the ER and not of the intermediate compartment J Cell Sci 1994 107 Pt 10 2719 2727 7876340 10.1242/jcs.107.10.2719

[B32] Guo W Qu F Xia L Guo Q Ying X Ding Z Identification and characterization of ERp29 in rat spermatozoa during epididymal transit Reproduction 2007 133 575 584 10.1530/REP-06-0301 17379652

[B33] He H Li J Weng S Li M Yu Y S100A11: Diverse Function and Pathology Corresponding to Different Target Proteins Cell Biochem Biophys 2009 55 117 126 10.1007/s12013-009-9061-8 19649745

[B34] Wong WS Zhao J Proteome analysis of chronically inflamed lungs in a mouse chronic asthma model Int Arch Allergy Immunol 2008 147 179 189 10.1159/000142040 18594147

[B35] Middleton E Jr Antiasthmatic drug therapy and calcium ions: review of pathogenesis and role of calcium J Pharm Sci 1980 69 243 251 10.1002/jps.2600690244 6244384

[B36] Ernster L Schatz G Mitochondria: a historical review J Cell Biol 1981 91 227s 255s 10.1083/jcb.91.3.227s 7033239 PMC2112799

[B37] Green DR Kroemer G The pathophysiology of mitochondrial cell death Science 2004 305 626 629 10.1126/science.1099320 15286356

[B38] McBride HM Neuspiel M Wasiak S Mitochondria: more than just a powerhouse Curr Biol 2006 16 R551 560 10.1016/j.cub.2006.06.054 16860735

[B39] Roth M Black JL An imbalance in C/EBPs and increased mitochondrial activity in asthmatic airway smooth muscle cells: novel targets in asthma therapy? Br J Pharmacol 2009 157 334 341 10.1111/j.1476-5381.2009.00188.x 19371343 PMC2707981

[B40] Trian T Benard G Begueret H Rossignol R Girodet PO Ghosh D Ousova O Vernejoux JM Marthan R Tunon-de-Lara JM Berger P Bronchial smooth muscle remodeling involves calcium-dependent enhanced mitochondrial biogenesis in asthma J Exp Med 2007 204 3173 3181 10.1084/jem.20070956 18056286 PMC2150973

[B41] Ozawa T Sako Y Sato M Kitamura T Umezawa Y A genetic approach to identifying mitochondrial proteins Nat Biotechnol 2003 21 287 293 10.1038/nbt791 12577068

[B42] Fernie AR Carrari F Sweetlove LJ Respiratory metabolism: glycolysis, the TCA cycle and mitochondrial electron transport Curr Opin Plant Biol 2004 7 254 261 10.1016/j.pbi.2004.03.007 15134745

[B43] Graham JW Williams TC Morgan M Fernie AR Ratcliffe RG Sweetlove LJ Glycolytic enzymes associate dynamically with mitochondria in response to respiratory demand and support substrate channeling Plant Cell 2007 19 3723 3738 10.1105/tpc.107.053371 17981998 PMC2174870

[B44] Pizzo P Pozzan T Mitochondria-endoplasmic reticulum choreography: structure and signaling dynamics Trends Cell Biol 2007 17 511 517 10.1016/j.tcb.2007.07.011 17851078

