# Supplementary material for: The early asthmatic response is associated with glycolysis, calcium binding and mitochondria activity as revealed by proteomic analysis in rats
Source: Respir Res. 2010 Aug 6;11(1):107. doi: 10.1186/1465-9921-11-107 (PMC2925830; doi:10.1186/1465-9921-11-107)
Supplement: Additional file 1 — Primer sequences for the selected genes and the semi-quantitative RT-PCR conditions. This table lists the primer sequences used to amplify the three genes of interest (S100A8, S100A11 and VDAC1) and the reference gene (β-actin) as well as the corresponding semi-quantitative RT-PCR conditions. [file 1465-9921-11-107-S1.DOC]

**Additional file 1 - Primer sequences for the selected gens and semi-quantity RT-PCR conditions.**

| Gene | Primer sequence | Product size (bp) | Annealing (ºC) | PCR cycles |
| --- | --- | --- | --- | --- |
| S100A8 | Forward 5’ATGGCAACTGAACTGGAGAA3’ | 227 | 58 | 32 |
|  | Reverse 5’ATCACCAACGCAAGGAACTC3’ |  |  |  |
| S100A11 | Forward 5’TACAGTGGGAAGGATGGAA3’ | 232 | 58 | 32 |
|  | Reverse 5’TCTGGGAAGTCTGGAGGAA3’ |  |  |  |
| VDAC1 | Forward 5’AGTGAACGGCAGTCTGGAAA3’ | 288 | 58 | 30 |
|  | Reverse 5’CAACCCTCATAGCCAAGCAC3’ |  |  |  |
| β-actin | Forward 5’CAACCTTCTTGCAGCTCCTC3’ | 555 | According to target gen | |
|  | Reverse 5’CCCTCATAGATGGGCACAGT3’ |  |  |  |
