# Supplementary material for: The early asthmatic response is associated with glycolysis, calcium binding and mitochondria activity as revealed by proteomic analysis in rats
Source: Respir Res. 2010 Aug 6;11(1):107. doi: 10.1186/1465-9921-11-107 (PMC2925830; doi:10.1186/1465-9921-11-107)
Supplement: Additional file 2 — The list of the differentially expressed proteins in the enriched gene ontology (GO) classes. This table lists the details of the GO classification including the enriched GO classes and corresponding differentially expressed proteins. [file 1465-9921-11-107-S2.DOC]

**Additional file 2 - The list of differentially expressed proteins of enriched Gene Ontology (GO) classes.**

| **GO ID^*^** | **Description^†^** | **Number of proteins** | **Identified proteins^‡^** | ***P*-value^§^** |
| --- | --- | --- | --- | --- |
| GO:0006096 | glycolysis(BP) | 6 | ENO3 PGK1 ENO2 GAPDH PGAM1 PKM2 | < 0.05 |
| GO:0006457 | protein folding(BP) | 5 | ERP29 HSPA8 PDIA6 CALR HSPA2 | < 0.05 |
| GO:0000166 | nucleotide binding(MF) | 10 | PEBP1 TUBA1A HSPA8 ATP1A1 HSPA2 ACTA1 PGK1 PSMC6 CKB ATP5A1 | < 0.05 |
| GO:0016787 | hydrolase activity(MF) | 5 | ATIC PSMC6 ATP1A1 PGAM1 ATP5A1 | < 0.05 |
| GO:0005509 | calciumion binding(MF) | 5 | S100A8 S100A11 PDIA6 CALR PRSS1 | < 0.05 |
| GO:0005829 | cytosol(CC) | 7 | PGK1 NANS_PREDICTED PSMC6 GAPDH CALR PGAM1 PKM2 | < 0.05 |
| GO:0005739 | mitochondrion(CC) | 7 | TUBA1A GAPDH HSPA2 PRSS1 CKB PKM2 ATP5A1 | < 0.05 |

* The unique identifier for the GO class (or term) being annotated.

† BP, biological process to which gene products contribute; MF, molecular function that describes gene products’ biochemical activities; CC, cellular component refers to the subcellular location where gene products are active and occur.

‡ Protein identities are designated with their standard Gene symbol or name.

§ Statistical significance analysis made by a Monte Carlo simulation procedure.
